# Mumtathil: Automatic PDPL compliance identification system of Arabic privacy policies documents

**DOI:** 10.1371/journal.pone.0351074

**Published:** 2026-06-10

**Authors:** Malak Mashaabi, Hend Al-Khalifa

**Affiliations:** Department of Information Technology, College of Computer and Information Sciences, King Saud University, Riyadh, Saudi Arabia; University of Lagos Faculty of Engineering, NIGERIA

## Abstract

In the digital era, the extensive collection and use of personal data by websites and applications has prompted various countries to implement data protection laws. The Kingdom of Saudi Arabia introduced the Personal Data Protection Law (PDPL) to regulate the handling of personal data. However, manually assessing compliance with these laws is time-consuming, and costly. This paper presents an automated system named Mumtathil (مُمتثِل) that evaluates the compliance of Arabic privacy policies with the PDPL using multi-label classification (MLC) techniques. Our experiments demonstrated that the Support Vector Machine (SVM) outperformed other machine learning models and transformer-based models like CamelBERT and AraBERT, achieving an F-score of 92%. The system addresses dataset imbalance by augmenting the Saudi Privacy Policy Dataset with recent privacy policies and synthetic data generated using ChatGPT. The Mumtathil tool simplifies compliance assessment, reducing time, cost, and effort while enhancing transparency and data protection in the digital landscape.

## 1. Introduction

In recent years, the rapid expansion of digital data has led to the widespread collection and use of personal information by websites and applications. This data often includes sensitive details like identification and credit card numbers. In response, many countries have generated laws to protect data and ensure users are informed about how their information is being used. These laws specify the types of personal data collected, how it is utilized, the measures for secure storage, and other related aspects, such as penalties for violations. Famous examples include the General Data Protection Regulation (GDPR), which regulates information privacy in the European Union [1], California Consumer Privacy Act (CCPA) which enhances privacy rights and consumer protection for residents of the state of California in the United States [2], and the Personal Information Protection Law (PIPL), which safeguards personal information rights and interests in China [3].

The Kingdom of Saudi Arabia (KSA) published the Personal Data Protection Law (PDPL), effective from September 17, 2023 [4]. A critical aspect of the PDPL is its emphasis on transparency and user rights. It mandates that entities must provide a clear privacy policy to their users before any data collection or processing occurs. This policy must explicitly inform users about their rights as outlined in the PDPL, ensuring they are fully aware of how their data is being handled. However, privacy policies present several challenges. Firstly, their length and complexity can make them difficult to understand. Ensuring compliance with these policies requires a thorough understanding of the functionalities of websites or applications and their associated legal obligations, which can be challenging for developers when drafting privacy policies that fully comply with data protection regulations [5]. Moreover, manually assessing whether a privacy policy complies with relevant laws is challenging due to the extensive time, effort, and cost required.

While full PDPL compliance requires normative legal judgment that considers contextual factors beyond textual analysis, automated category detection serves as a foundational tool to support compliance reviewers in identifying relevant policy sections. Our work focuses specifically on detecting the presence of PDPL-related categories within privacy policies—a necessary first step that helps legal experts and compliance officers efficiently locate and assess relevant information.

To address these challenges, this paper presents an automated system named Mumtathil (مُمتثِل) that evaluates the compliance of Arabic privacy policy documents with the PDPL using multi-label classification (MLC) techniques. Our experiments demonstrated that the Support Vector Machine (SVM) outperformed other machine learning (ML) models, such as Logistic Regression (LR) and Random Forest (RF), as well as transformer-based models like CamelBERT and AraBERT, achieving an F-score of 92%.

This study makes several significant contributions in the domain of privacy policy compliance analysis, particularly in the context of Arabic data protection regulations. The key contributions of this work are as follows:

- Reconstructed and expanded the Saudi Privacy Policy Dataset by incorporating recent privacy policy documents and augmenting it with synthetic data generated using ChatGPT. This enhancement addresses dataset imbalances and improves coverage for compliance classification.
- Conducted an extensive evaluation of traditional machine learning models, including SVM, RF, and LR, to assess their effectiveness in analyzing privacy policy compliance.
- Explored the performance of Arabic transformer models, such as CamelBERT and AraBERT, and introduced an overlap technique to handle the challenges posed by lengthy privacy policy documents.
- Provide a practical solution by developing a web-based tool, Mumtathil, designed to automate the analysis of Arabic privacy policy documents for compliance with Saudi PDPL.

The remainder of this paper is structured as follows: Section 2 provides background on information privacy, the Saudi PDPL, and reviews the related literature. Section 3 presents the methodology, including dataset preparation, preprocessing, modeling, evaluation, and system design. Section 4 presents the experimental results. Section 5 discusses the findings, including limitations. Finally, Section 6 presents the conclusion and future work.

## 2. Background and related work

This section provides the theoretical and empirical background for this study. It begins with foundational concepts in information privacy and text classification, then reviews related work on automated privacy compliance assessment, multi-label classification in Arabic, and long-document classification techniques.

### 2.1. Information privacy

Individuals place high importance on the confidentiality of their private lives and on safeguarding their personal information. Personal information is defined as any data that can identify someone either directly or indirectly, including details like name, date of birth, credit card information, and email address [6]. In relation to this, information privacy is the right to control this personal information, allowing individuals, groups, or institutions to decide how and when their data is shared [7]. This privacy has been threatened by recent technological advances, which reduce control over personal data.

KSA published its own PDPL on September 24, 2021, which was supposed to be effective on March 23, 2022, but its effect was postponed to September 17, 2023 [8].

The purpose of the PDPL is to protect individual privacy by regulating the gathering, use, disclosure, and retention of personal data. There are 43 articles in the PDPL that deal with definitions, the law’s scope of application, data processing standards, data subject rights, and penalties for breaking the law. Additionally, it outlines the legal rights of processing residents’ personal data by all entities either inside or outside KSA.

The key PDPL principles are listed below [4]:

1) *User Consent:* Specifies the importance of user consent in handling their personal information.
2) *Data Collection and Processing:* Specifies the importance of clarifying the purpose, method, and content of data that is collected.
3) *Data Retention:* Specifies the importance of destroying personal data once the original purpose of its collection is fulfilled.
4) *Data Protection:* Specifies the importance of keeping personal data safe.
5) *Data Sharing:* Specifies the importance of avoiding sharing, transferring, or disclosing personal data to any other parties.
6) *Data Rectification:* Specifies the importance of users’ rights to update their personal data held by any public or private entity.
7) *Data Destruction:* Specifies the importance of the users’ rights to request the deletion of their personal data.
8) *Data Access:* Specifies the importance of the users’ rights to view and obtain a copy of their personal data.
9) *Advertisements:* Specifies the importance of users’ approvals to use their personal data by communication channels in sending promotions. It also specifies the user’s right to stop receiving such advertisements.
10) *Breach Notification:* Specifies the importance of notifying the competent authority and data owner of leakage or corruption of personal data.
11) *Responsibility:* Specifies the importance of entities responsibility to adhere to the PDPL and data processing practices.

### 2.2. Text classification in natural language processing

Natural Language Processing (NLP), a branch of Artificial Intelligence (AI), focuses on enabling computers to understand and process spoken and written language in a manner similar to human understanding [9].

One of the essential tasks in NLP is text classification, which involves extracting features from raw text data and predicting categories based on those features. Text classification is widely used in sentiment analysis, topic labeling, spam detection, and intent detection. There are two main types of text classification [10]: Single-Label Classification (SLC) and Multi-Label Classification (MLC). SLC is the standard classification approach that assigns each text to a single predefined label. It might be a binary classifier where text is categorized into two distinct classes, like classifying tweets as positive or negative, or a multi-class classifier where text is categorized into only one of several classes with each class being independent of the others such as classifying emails into classes like spam, other, or non-spam.

On the other hand, MLC categorizes text into one or more classes simultaneously, with each class potentially dependent on others. For instance, a news article given the “science” label may also be assigned labels like “education” and “technology” at the same time [11].

MLC is a challenging task in NLP due to problems including imbalanced data distribution, incorrect or missing labels, and increased computational complexity as the number of labels increases [12]. To address these challenges, ML techniques tailored for MLC have been developed. These techniques can be broadly categorized into two approaches:

1) *Flat Classification* [10]: This approach handles each label independently and categorizes them at a single level. It is particularly useful when there are no relationships between labels. Flat classification can be further divided into two techniques:
  - Problem Transformation Technique: This technique transforms a multi-label issue into multiple single-label problems. The Binary Relevance (BR) method is an example of this approach, where traditional single-label classification algorithms, such as SVM, LR, and RF can be applied effectively.
  - Algorithm Adaptation Technique: This technique modifies existing single-label algorithms to make them compatible with multi-label data, such as Multi-Label Decision Tree (ML-DT).
2) *Hierarchical Classification* [13]: This approach is beneficial when there are relationships between labels, handling categorization at multiple levels.

Several traditional ML algorithms have been widely used for text classification tasks. Some of the most common ones include:

1) *Support Vector Machines (SVM):* SVM is a discriminative classifier that aims to find the optimal hyperplane separating different classes in a high-dimensional space. It is particularly effective for text classification because it handles high-dimensional and sparse data [14].
2) *Logistic Regression (LR):* LR is a type of probabilistic classification model that makes predictions about categorical dependent variables by using one or more features as predictors [15].
3) *Random Forest (RF):* RF is an ensemble learning method that combines multiple decision trees. Each tree is trained on a random subset of features and samples, and the final prediction is obtained by aggregating the predictions of individual trees [16].

While these traditional ML algorithms have proven effective in various text classification domains, fine-tuning transformer models have shown remarkable performance improvements compared to these approaches. Pre-trained transformer models capture rich linguistic knowledge and contextualized representations, which can be effectively transferred to specific classification tasks [17].

Several transformer-based models have been specifically developed for Arabic language processing, such as:

1) *AraBERT:* is a pre-trained Bidirectional Encoder Representations from Transformers (BERT) model for Arabic language understanding. It has been trained on a large corpus of Arabic text and has achieved state-of-the-art performance on various Arabic NLP tasks, including text classification [18].
2) *CamelBERT:* is a collection of BERT models that have been pre-trained on Arabic texts, containing various sizes and variants. This includes pre-trained language models for Modern Standard Arabic (MSA), dialectal Arabic (DA), and classical Arabic (CA), as well as a model that is pre-trained on a combination of these three [19].

These Arabic-specific transformer models have opened new possibilities for improving the performance of Arabic text classification systems by leveraging the power of pre-trained language representations. However, the self-attention mechanism is particularly effective for sequences with a token count not exceeding 512, as exemplified by models like BERT. As the text length increases, the performance of these models tends to decrease. Moreover, the length of the text leads to significantly more computational complexity, making the representation of long documents a challenging task in NLP, even with transformer-based models.

### 2.3. Automated assessment of data privacy compliance

In recent years, many researchers have focused on assessing compliance with regional laws. The GDPR represents most of the research. This is not unexpected considering that the GDPR was introduced in 2018 compared to other regulations. Researchers have employed a wide variety of methodologies, ranging from semantic analysis to ML and deep learning techniques. These diverse approaches demonstrate the evolving strategies to address the challenges of privacy policy compliance. Elluri et al. [20] developed an advanced framework for evaluating the similarity between GDPR regulations and cloud privacy policies, addressing the limitations of traditional text similarity methods which often ignore the deeper meaning and context of words. Their approach combined Semantic Web and NLP techniques, enhancing the comparison of short-text policy documents. The effectiveness of their framework was demonstrated through its application to 20 GDPR-compliant cloud privacy policies, displaying its capability to measure semantic similarity between GDPR regulations and cloud privacy policies, and in the construction of a knowledge graph. In a subsequent study, Elluri et al. [21] introduced a framework to assess web service policy compliance with GDPR classes, as these policies are typically shorter than regulation texts, making manual compliance challenging due to requiring significant human effort to understand and implement. They employed traditional ML methods (Naive Bayes, LR, SVM, RF) and deep learning models (Convolutional Neural Networks (CNN), Long Short-Term Memory (LSTM), BiLSTM) for multi-class classification. The results showed that the BiLSTM outperformed the NB, LR, RF, and SVM machine learning models, as well as the CNN and LSTM deep learning models, with an F-score of 78%. In contrast, Amaral et al. [22] introduced an automated solution, Data Processing Agreements sEmantic fRamE-based Compliance cHecking Against GDPR (DERECHA), to verify the compliance of Data Processing Agreements (DPA) with GDPR. They utilized semantic frames to automatically capture the conceptual structure of sentences, including the roles of various entities and their interrelationships. Their method was tested on 54 real DPAs and achieved an average precision of 89.1%, a recall of 82.4%, and an accuracy of 84.6%.

In the context of other regulations, Asif et al. [23] examined the Pakistan Data Protection Act (PDPA) by providing the foundation for creating a tool designed to automatically assess the privacy policies of Pakistani websites, determining their compliance with both GDPR and PDPA. They evaluated 120 Pakistani websites using the Term Frequency – Inverse Document Frequency (TF-IDF) method for vectorization and tested four ML classifiers: SVM, LR, NB, and K-Nearest Neighbors (KNN), with the SVM achieving a high accuracy of 97.7% in identifying compliance.

Furthermore, Mashaabi et al. [24] established a baseline for assessing the compliance of Saudi websites with the PDPL by constructing an annotated corpus of 1,000 websites. They used the TF-IDF and Word2Vec methods for representation the text and tested three ML techniques (LR, NB, SVM), Deep Learning techniques (LSTM, Feed-Forward Neural Network (FFNN)), and Transformers (ARABERT, MARBERT, CamelBERT). The result showed that the MARBERT and CamelBERT models achieved the best performance with a micro-average F-score of 93%.

### 2.4. Multi-label text classification in Arabic language

The MLC in the Arabic language presents unique challenges due to its distinct characteristics and the high dimensionality of its representation [11]. In 2023, Alzanin et al. [25] recognizing the problem of high dimensionality in representation, introduced a novel approach by employing a genetic algorithm for effective feature selection, combined with ensemble learning. This ensemble model merges predictions from multiple classifiers to produce more accurate results. When comparing ensemble methods, they found the Extra Trees Classifier (ETC) to be particularly effective, over the LR and RF in performance. Specifically, ETC achieved an F-score of 65.62% without feature selection and 68.76% with it. Moreover, Rahmana and Baawi [26] proposed a method to enhance MLC. Their approach adapted traditional ML algorithms to address multi-label challenges. They utilized BR to split the multi-label task into multiple single-label problems. Alternatively, the label powerset (LP) method transformed the multi-label problem into a multi-class one by creating unique classes for each label combination. The chi-square feature selection method was used to enhance the efficacy of the proposed method. Among the classifiers evaluated, LR, RF, and Multinomial NB; BR with LR emerged as the top performer, achieving an average micro-Recall of 86%. Alternatively, Al Jedani et al. [27] approached Arabic text classification from a hierarchical perspective with their Hierarchical Multi-label Arabic Text Classification (HMATC) model. This machine learning-based approach involved crucial stages like feature extraction using Information Gain and Gain Ratio, and feature selection via classifiers like LP and BR. Their methodology was tested on the Fatawa dataset. For comparison, HMATC was evaluated against state-of-the-art models which are Fatwa and Classifier Chains models, as well as the baseline classifier which is SVM as the base classifier for BR, CC, and LP models. The result showed BR achieved the best results with a micro-averaged F-measure of 85.77% and a Hamming loss of 0.0043. This was marginally better compared to their proposed HMATC model, which achieved a micro-averaged F-measure of 85.33% and a Hamming loss of 0.0045.

### 2.5. Long Document Classification

Transformer techniques have demonstrated effectiveness in semantic analysis and text classification, but their dependence on the self-attention mechanism can increase complexity when working on long documents [28]. Researchers’ efforts range from refining existing models such as BERT to developing new Transformer architectures like the Longformer. The focus is on enhancing Transformer-based methods to support documents exceeding 512 tokens.

One of the most popular transformers, BERT, the major issue associated with BERT is the maximum input sequence length is constrained to 512 tokens. Researchers have addressed this issue by exploring strategies such as chunk segment selection, document truncation, and key sentence selection [28].

Ding et al. [29] addressed the token limitations of BERT by introducing the CogLTX framework. This approach employs two BERT models: a judge model and a reasoner model. The judge model, through its training, identifies key sentences. These sentences are then feeding into the reasoner model to execute the desired task. To evaluate the effectiveness of CogLTX, Ding et al. applied it to various applications. One significant experiment involved MLC using the Alibaba dataset, which comprises 30,000 articles. The CogLTX technique demonstrated its outperforming SVM, Bi-LSTM, TextCNN, and sliding window techniques, achieving a 97.8% Micro-F-score.

Furthermore, Beltagy et al. [30] proposed a new transformer called the Longformer. What sets the Longformer apart is its innovative attention mechanism that linearly scales with sequence length, enabling it to process up to 4096 tokens. This is achieved by combining local windowed attention with task-driven global attention. The local attention allows Longformer to focus on the most relevant parts of the sequence, while the global attention ensures the capture of long-distance dependencies. To evaluate the Longformer’s performance, Beltagy et al. applied it to a range of tasks, including document classification using the IMDB dataset, which contains 654 documents. They compared the Longformer’s performance with that of the RoBERTa transformer, finding that the Longformer achieved accuracy rate of 95.7%.

Dai et al. [31] undertook an additional experiment involving the Longformer Transformer. They noted that in the IMDB dataset, just 15% of the examples exceed 512 tokens in length, and the dataset itself includes only 645 documents. Their research provided a comparison between different Transformer-based Long Document Classification (TrLDC). The focus was primarily on sparse attention and hierarchical transformers. For a comparison of different TrLDC methods, they utilized four datasets, including Medical Information Mart for Intensive Care (MIMIC-III) and European Court of Human Rights (ECtHR), both of which are multilabel datasets. The results showed that on MIMIC-III and ECtHR datasets, both the Longformer and hierarchical Transformers outperformed baseline models restricted to handling up to 512 tokens which included First, Random, and Informative.

From the presented literature, studies commonly utilize ML to verify compliance with target regulations [21,23,24], using metrics like Precision, Recall, and F-score for evaluation [21,22,24]. However, a limitation in dataset size was observed, with the smallest dataset involving only 20 documents [20] and the largest involving 3,000 documents [21]. Turning to long document classification, the focus was on enhancing Transformer-based methods to support documents exceeding 512 tokens, with performance typically evaluated using metrics such as Micro-F1 and Accuracy [29,31]. Additionally, the unique linguistic features of the Arabic language have led to specialized research [25–27]. In all these areas, performance is assessed using metrics like Precision@K, Recall@K, Micro F-score, and Hamming Loss.

From a theoretical standpoint, the Contextual Integrity framework proposed by Nissenbaum [32] provides an important normative lens for privacy analysis. This framework holds that privacy is respected when information flows match the norms of the context in which data was originally disclosed. While Contextual Integrity is a powerful basis for assessing legal adequacy, applying it requires interpretive judgment that goes beyond automated text classification. Our work is positioned as a complementary, first-step tool: by automating PDPL category detection, we help compliance reviewers efficiently identify which normative dimensions require further legal examination, rather than replacing human judgment. In parallel, recent work has demonstrated the potential of large language models for privacy policy analysis. Studies leveraging GPT-4 and related models have explored automated compliance checking against GDPR [33] and privacy policy summarization and gap detection [34]. Unlike these generative approaches, our system employs discriminative classification using Arabic-specific models (SVM, AraBERT, CamelBERT) trained on a purpose-built PDPL dataset, addressing a gap in Arabic regulatory NLP where such resources and benchmarks are scarce.

In this paper, we aim to automate the analysis of Arabic privacy policy documents for compliance with the PDPL. To our knowledge, only one study has addressed this area [24], using multi-class classification, which may not be practical for real-world applications as it requires segmenting privacy policies. Users typically need to analyze entire documents without segmentation for compliance checks. Additionally, collecting datasets during the initial PDPL implementation was challenging, as many privacy policies did not fully address all requirements, impacting model training. To address these gaps, we aim to augment the dataset with recent privacy policies and develop a web-based solution utilizing MLC suitable for analyzing long documents. This approach aligns with practical needs, allowing users to efficiently assess complete privacy policies for PDPL compliance.

## 3. Methodology

The methodology of this study follows an NLP pipeline to analyze Arabic privacy policy documents for compliance with the Saudi PDPL. The workflow includes dataset preparation, data cleaning and preprocessing, text representation, model training, evaluation, and system development.

**Fig 1:**
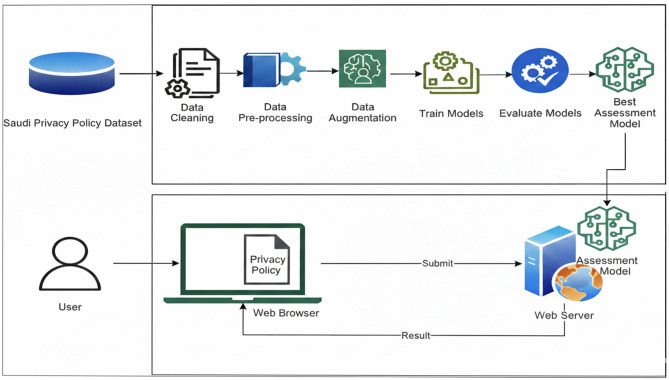
System development framework for the Mumtathil tool.

The overall system framework is illustrated in Fig 1.

### 3.1. Dataset preparation

The Saudi Privacy Policy Dataset by Al-Khalifa et al. [35] is acquired from various sources such as the Saudi Central Bank, the Saudi Arabia National United Platform, and the Council of Health Insurance. This dataset ensures diversity by encompassing seven sectors illustrated in Table 1.

**Table 1:** Description of seven sectors represented in the Saudi Privacy Policy Dataset.

Sector	Description	Websites Number
News	Includes websites for news, articles, etc.	19
Healthcare	Includes the websites of hospitals, clinics, health insurance companies, etc.	27
Educational	Includes websites of universities, institutes, academies, etc.	42
Finance	Includes banks and all financial-related websites	52
Government	Includes all governmental entities denoted by the (gov) extension	80
Other	Includes any website that does not belong to the remaining categories such as technical companies	155
E-commerce	Includes all commercial websites, online stores, etc.	625

**Fig 2:**
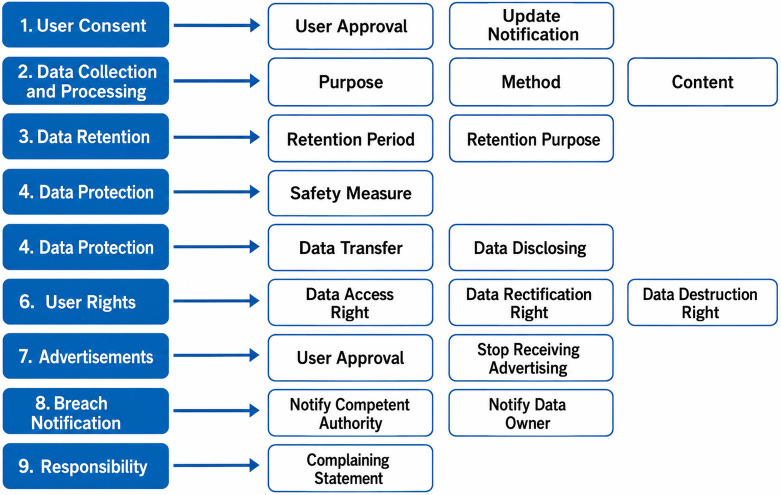
Categorization of PDPL principles in the Saudi Privacy Policy Dataset.

Collected in December 2022, the dataset contains a total of 1,000 privacy policies. The three people who performed the annotation to assign labels hold Bachelor’s degrees in the field of Information Technology and have a native Arabic language background. Cohen’s Kappa has been used to measure the agreement between them, and the result showed almost perfect agreement between each pair of annotators, with an average of 0.953. The annotation process resulted in the segmentation of the privacy policies into 4,638 different lines. Each line has been labeled with a relevant principle from the PDPL, totaling 498,808 tokens and a size of 5.73 MB. The PDPL principles are organized into nine categories, as illustrated in **Fig 2**. Additionally, Al-Khalifa et al. added a new category “tenth category” to involve any text in privacy policy documents that do not align with the PDPL principles, such as rights pertaining to children. However, this category was excluded from our study as it does not represent a principle defined within the Saudi PDPL.

#### Length and characteristics of labels.

**Fig 3:**
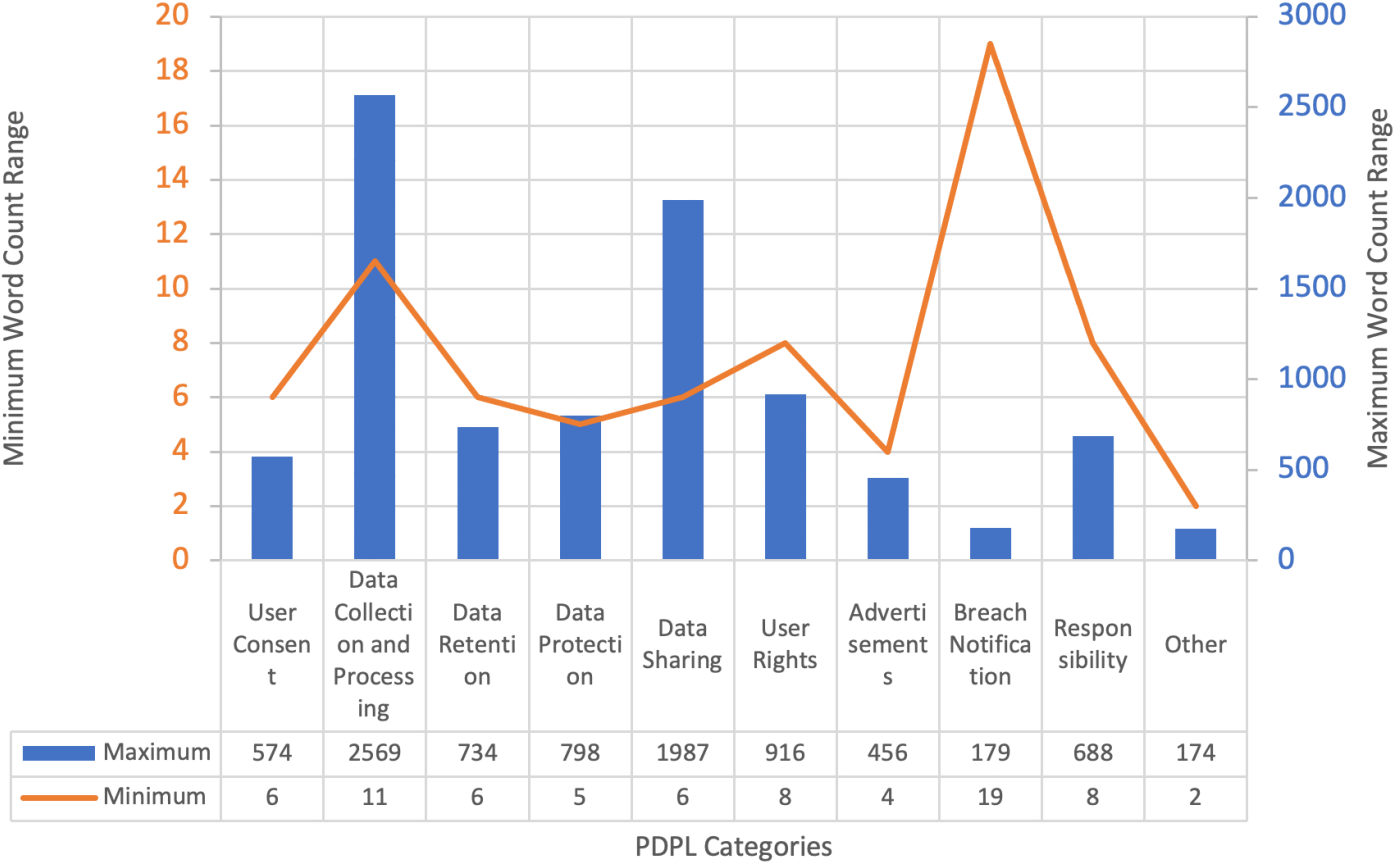
Differences in text length: maximum vs. minimum word counts by category.

An examination of text length across labels, as shown in Fig 3, reveals variation in text length across categories, which reflects differences in content complexity and supports the need for robust text representation methods.

**Fig 4:**
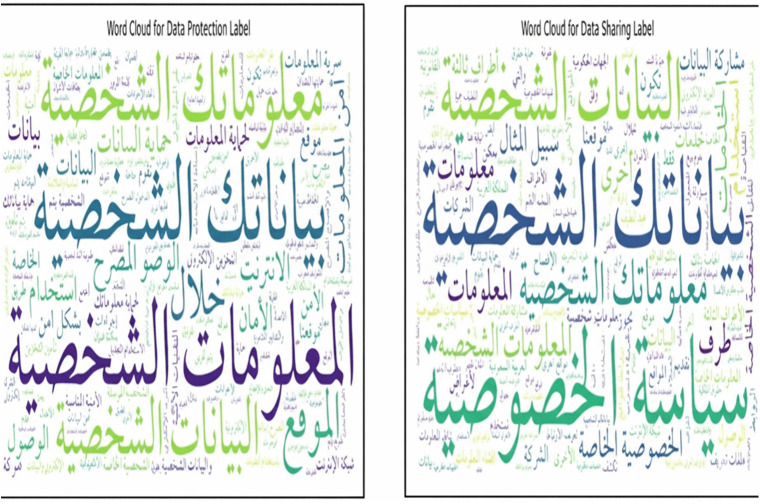
Word cloud representation of the Data Protection Label and Data Sharing Label.

To better understand the vocabulary patterns, word clouds were created for each label, visually representing word frequency where larger text indicates higher occurrence, as illustrated in Fig 4. Notably, the word clouds for ‘Data Protection’ and ‘Data Sharing’ labels revealed significant similarities and extensive vocabulary overlap between these two categories, which motivated the decision to merge these labels in subsequent experiments and evaluate the impact of this consolidation on model performance.

The Saudi Privacy Policy Dataset was collected during the PDPL’s implementation period, with enforcement postponed to September 17, 2023. Consequently, many privacy policies at that time did not address certain PDPL categories, resulting in imbalanced sector representation.

To address this, we manually collected 100 additional privacy policies in August 2023, focusing on underrepresented sectors: Healthcare, Educational, and Other. Table 2 shows the updated distribution across sectors.

**Table 2:** Updated count of privacy policies by sector in Saudi Privacy Policy Dataset.

Sectors	Before Expansion	After Expansion
E-commerce	625	629
Government	80	85
Finance	52	60
Healthcare	27	54
Educational	42	71
News	19	No Change
Others	155	182
**Total**	**1,000**	**1,100**

The annotation process followed the same mechanism as the original dataset, utilizing the same annotator to ensure consistency.

**Fig 5:**
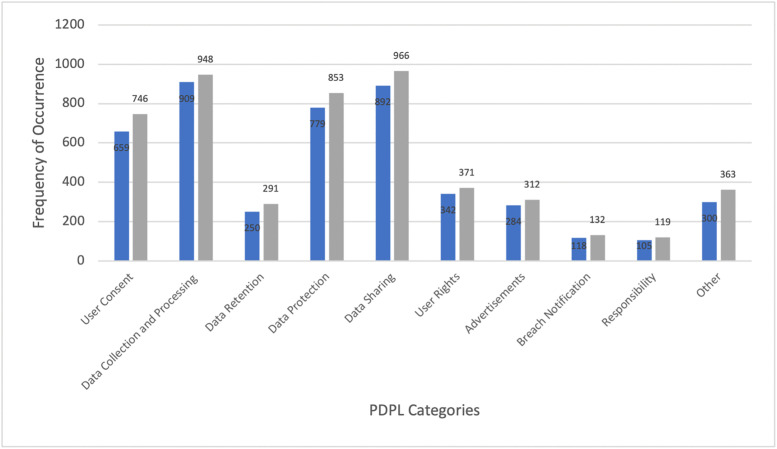
Distribution of labels in the Saudi Privacy Policy Dataset before and after augmentation.

For MLC implementation, we reconstructed the dataset and employed the BR method, where each privacy policy occupies a single row with binary labels for each PDPL category (1 = compliant, 0 = non-compliant). Fig 5 presents the label distribution before and after expansion, highlighting the persistent class imbalance inherent in MLC tasks.

Analysis of compliance patterns showed that most privacy policies complied with only four PDPL categories, indicating inadequate overall compliance. Specifically, 317 privacy policies complied with four categories, followed by 239 policies complying with five categories, and 207 policies complying with three categories. Notably, only two privacy policies achieved full compliance with all nine PDPL categories.

Because most privacy policies were compliant with four PDPL categories, we conducted a detailed analysis of the dataset to explore the four combinations of categories that frequently appear together in privacy policies.

Our findings showed that the categories of User Consent, Data Collection and Processing, Data Protection, and Data Sharing were commonly found together, appearing in 480 privacy policies. This was followed by the combination of Data Collection and Processing, Data Protection, Data Sharing, and User Rights categories, which appeared in 260 privacy policies.

**Fig 6:**
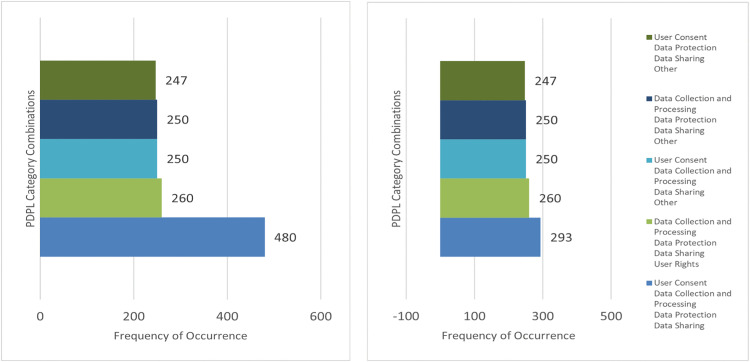
Top four most frequent PDPL category combinations in the updated Saudi Privacy Policy Dataset before and after applying the under-sampling technique.

Due to the high frequency of the combination of User Consent, Data Collection and Processing, Data Protection, and Data Sharing, we decided to adjust its representation to balance the distribution of the other grouped labels. We applied random under-sampling technique to these labels by randomly selecting a subset of instances from the majority class to decrease its size while keeping all the data in the minority class. This process helps to balance the distribution of the grouped labels, as shown Fig 6.

#### Synthetic data creation using large language model.

Synthetic data creation involves generating artificial data that closely mimics real-world data. This practice is becoming increasingly popular in the fields of AI, ML, and data science [36]. The reason behind the increase in its usage is several advantages, particularly its capacity to overcome challenges associated with real-world data. For example, by producing data for minority classes, synthetic data can address dataset imbalance and enhance the performance of ML models [37].

In this paper, we utilized the capabilities of ChatGPT [38], introduced by OpenAI on November 30, 2022, to tackle the data imbalance challenge. ChatGPT provides the flexibility to customize conversations to meet specific criteria such as length, format, style, detail, and language. We created a custom version of ChatGPT named ‘Privacy Policy Generator’ to generate detailed Arabic privacy policies, focusing on the minority categories in the Saudi Privacy Policy Dataset. To cover each category, we included clear definitions for them within the category name. Employing Few-Shot Learning techniques, we provided 5–10 examples per category, which significantly helps guide the model’s generation process accurately and effectively. Moreover, to promote diversity among the generated policies, we introduce an additional instruction directing the Privacy Policy Generator to omit one category in each privacy policy. Through iterative prompt refinement, we optimized the generation process. Initial prompts such as ‘Write an Arabic privacy policy’ provided basic outputs, which we progressively improved by adding specific directives like ‘Add more details in each section.’ We experimented with generating multiple policies per prompt, reaching up to 40 policies per generation. However, this resulted in decreased length and detail for each policy. Finally, we limited generation to 20 privacy policies per prompt to maintain adequate quality and detail in each document.

**Fig 7:**
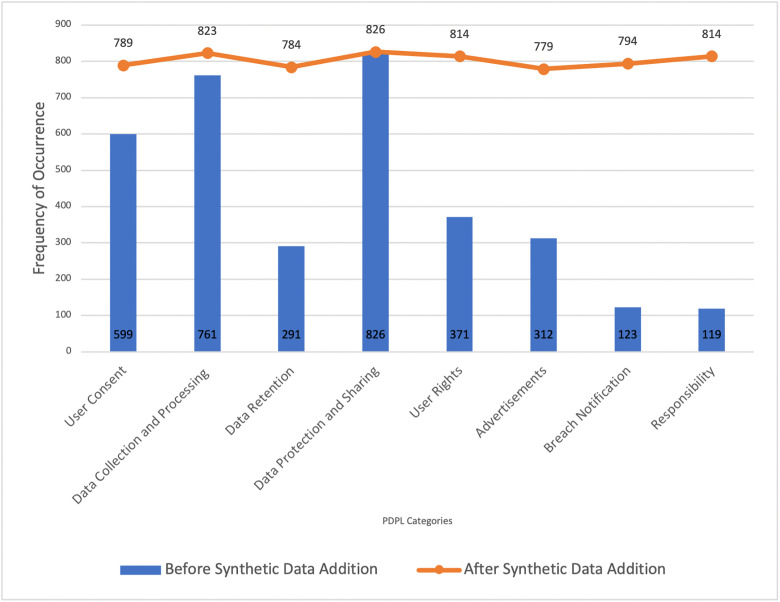
Distribution of labels within the Saudi Privacy Policy Dataset before and after adding the synthetic data.

To monitor category distribution and ensure dataset balance, we performed validation of the synthetic data concurrently with its generation. The validation process involved three individuals with relevant educational backgrounds. The first validator categorized each privacy policy according to PDPL categories. The second validator reviewed these initial labels to confirm accuracy. In cases of disagreement, a third validator served as an arbitrator. This structured approach enhanced the integrity and correctness of the synthetic data, minimizing errors and biases to produce a high-quality and balanced dataset. Fig 7 illustrates the distribution of labels within the dataset, both before and after adding the synthetic data, highlighting the expanded dataset.

#### Dataset balancing assessment.

To validate that the updated label distribution was balanced, we employed three widely used statistical measures:

- *Range* [39]: This is defined as the difference between the counts of the most and least frequent labels. In our analysis, the range was calculated to be 47 (826–779), presenting a limited difference between label frequencies.
- *Imbalance Ratio (IR)* [40]: IR is the most frequently used to assess the degree of imbalance in a dataset. This ratio compares the frequency of the most frequent label to that of the least frequent label. IR close to 1, indicates a relatively balanced dataset. The larger the IR indicate the larger the imbalance extent of the dataset. Our dataset showed an imbalance ratio of 1.06 (826/ 779), falling within the balanced range, which suggests a relatively even distribution among the labels.
- *Shannon Entropy* [41]: This measure calculates the randomness or uncertainty in the label distribution by using the following equation:

$$\begin{array}{c} H=-\sum p(x)\text{log}(\text{p}(x)) \end{array}$$

The higher values indicate a more uniform distribution. Our dataset’s entropy was approximately 2.82, indicating a high degree of randomness. The maximum Shannon Entropy achievable with our dataset’s label frequencies is 3. Based on these statistical values, we proceed to the next phase of our project, which involves cleaning and preprocessing the data to prepare it for representation and modeling. The final dataset comprises 1,888 privacy policies, each labeled with relevant PDPL principles, totaling 458,639 tokens and a size of 5.61 MB (see S1 Table).

### 3.2. Data cleaning and preprocessing

We applied systematic text preprocessing to prepare the Arabic privacy policies for classification. The preprocessing pipeline consisted of two main stages: cleaning and normalization.

#### Text Cleaning.

We employed regular expressions to remove English letters, numbers, symbols such as “@, -, *,.,!, etc”, blank rows, empty lines, and redundant spaces.

#### Stop Words Removal.

We removed frequently occurring words that carry minimal semantic value using the ‘nltk’ Arabic stop words list [42], supplemented with custom stop words that frequently appeared in the text but did not carry significant meaning in the context of privacy policies (e.g., أولا, ثانيا, البند, الأول). Arabic numerals (٠-٩) were also removed, as they carry no semantic value for classification.

#### Text Normalization.

To standardize character variations, we applied the following transformations:

- Mapping all variations [إ أ ٱ آ ا] to a standard form [ا].
- Converting [ؤ و} to [و}.
- Converting [ئ ى} to [ي}.
- Replacing [ة} with [ه}.
- Removing diacritization.

### 3.3. Text representation and modeling

Text was represented using TF-IDF, a common technique in studies focusing on Arabic multi-label classification [23,24]. In addition, we explored Word2Vec as an alternative text representation method.

For classification, we used machine learning models, including SVM, RF, and LR, to establish baseline performance.

In addition, we evaluated transformer-based models, including AraBERT and CamelBERT, to explore contextual representations for Arabic text. Since we were dealing with long privacy policy documents, and these transformers have a maximum input length of 512 tokens per chunk, the stride parameter allowed us to handle these lengthy texts effectively. By setting the stride, we ensured that the tokenization process created overlapping segments. This overlap strategy is designed to ensure that no important information is lost when segmenting long documents into manageable chunks.

### 3.4. Evaluation and experimental setup

Model performance was evaluated using precision, recall, F1-score, and hamming loss, which are commonly used metrics in multi-label classification tasks. In addition, confusion matrices were used to provide a detailed analysis of model predictions across different PDPL categories.

To ensure robustness and prevent overfitting, 7-fold cross-validation was applied across all ML models.

The experiments for this MLC task were conducted using Google Colab [43], a hosted Jupyter Notebook service that provides free access to processing power, including Graphics Processing Units (GPUs) and Tensor Processing Units (TPUs), without requiring local setup. The experiments were run on a TPU with a high RAM runtime configuration to speed up training times and allow for more complex model experimentation. Hyperparameter tuning was performed for each model, and the optimal settings are reported in Table 3.

**Table 3:** Hyperparameter settings for all models and representations.

Hyperparameter	TF-IDF			Word2vec
	SVM	RF	LR	
KFold Cross-Validation	7	7	7	7
Random State	42	42	42	42
Shuffle	True	True	True	True
Solver	N/A	N/A	liblinear	N/A
n_estimators	N/A	500	N/A	N/A
Probability	True	N/A	N/A	N/A
vector_size	N/A	N/A	N/A	300
window	N/A	N/A	N/A	10
min_count	N/A	N/A	N/A	1

For transformer models (CamelBERT and AraBERT), hyperparameters were carefully set to optimize performance and training efficiency, as illustrated in **Table 4**.

**Table 4:** Hyperparameter settings for transformer models.

Hyperparameter	Value
MAX_LENGTH	512
STRIDE	100
Batch Size	16
Learning Rate (lr)	5e-5
Loss Function	BCEWithLogitsLoss
Probability Threshold	0.5
Sigmoid Function	torch.sigmoid(logits)
Number of Epochs	3
Training Set	0.8
Testing Set	0.2
Average Evaluation Metrics	Micro

### 3.5. System Design and Implementation

#### System Architecture.

The tool allows users to upload privacy policies in either plain text or PDF format. Upon submission, the document is processed using a trained MLC model, identified as the best-performing model during the experimental evaluation. The model serves as the core classification component, analyzing the document and generating compliance results across the nine PDPL categories.

#### Compliance Assessment.

The tool employs a binary classification approach for each PDPL category, marking it with a checkmark (✓) if at least one aspect is detected in the document, or a cross mark (✗) if no aspects are found.

The tool assigns a compliance rank based on the number of main categories adhered to. The ranking system is categorized into three levels: ‘Weakly Compliant’ for documents adhering to 1–3 categories, ‘Moderately Compliant’ for those complying with 4–6 categories, and ‘Mostly Compliant’ for documents that meet 7–8 categories.

To provide a more accurate assessment, the compliance percentage is calculated using a weighted approach that accounts for the varying complexity of each PDPL category. Each category is assigned a weight based on the number of aspects it contains. For example, the Advertisements category contains two aspects: user approval to receive advertisements and the choice to stop receiving advertisements. The weight of this category is calculated by multiplying it by two. This weighting ensures that categories with more detailed requirements have a proportionally greater impact on the overall compliance score. The compliance percentage is calculated as:


$$\begin{array}{c} Compliance\;Percentage=\;(\Sigma \;W\_c)\;/\;(\Sigma \;W\_t)\;*\text{ 100} \end{array}$$

Where W_c is the weight of compliant categories and W_t is the weight of all categories. This approach ensures that the compliance percentage accurately reflects the importance of categories’ adherence to the PDPL principles.

#### User Interface.

**Fig 8:**
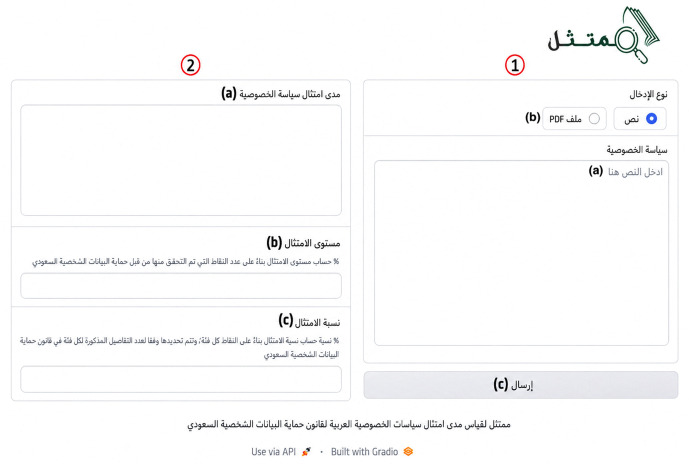
User Interface of the Mumtathil Tool: [1] Input Components: (a) Textbox for plain text input, (b) PDF upload option, (c) Submit Button [2] Output Components: (a) Classification results, (b) Compliance rank, (c) Compliance percentage.

As illustrated in Fig 8, the interface design emphasizes simplicity, consistency, and clarity in accordance with established UI design principles [44]. The interface is divided into two main sections: [1] Input components, including a textbox for plain text entry, a PDF upload option, and a submit button; and [2] Output components, displaying classification results for each PDPL category, the overall compliance rank, and the calculated compliance percentage. The tool supports Arabic language throughout, ensuring accessibility for the target user base.

## 4. Results

The experimental evaluation was conducted in four phases to assess the performance of the proposed approach and analyze the impact of dataset augmentation, label merging, and model selection.

### 4.1. Phase 1: Baseline models performance

The performance of each model in the first phase, which was the baseline with the imbalanced dataset is presented in Table 5.

**Table 5:** Classifier performance during the baseline phase with the imbalanced dataset, evaluating the initial effectiveness of different models before data augmentation.

Text Representation	Models	Precision	Recall	F-score	Hamming Loss
**TF-IDF**	LR	0.84	0.85	0.84	0.15
	RF	0.86	0.87	**0.87**	**0.13**
	SVM	0.85	0.88	0.86	**0.13**
**Word2vec**	LR	0.84	0.83	0.84	0.16
	RF	0.87	0.85	0.86	**0.13**
	SVM	0.84	0.83	0.83	0.16

The results showed that TF-IDF generated better results overall compared to Word2Vec. This indicates that the importance of a word in a document as measured by TF-IDF is more predictive than the contextual relationships between words captured by Word2Vec. In summary, the best performance of TF-IDF suggests that recognizing the frequency and uniqueness of terms is more valuable than how likely a word is to appear near another word.

Among the models, RF with TF-IDF achieved the best performance (F-score: 0.87), followed by SVM with TF-IDF (F-score: 0.86). Word2Vec representations yielded relatively lower performance, indicating that the semantic features were not as effectively utilized by the models as the keyword-based features from TF-IDF. LR showed consistent performance across both representations (F-score: 0.84). In terms of Hamming Loss, RF with both TF-IDF and Word2Vec, as well as SVM with TF-IDF, achieved the lowest values (0.13), indicating fewer prediction errors across labels compared to other models.

**Fig 9:**
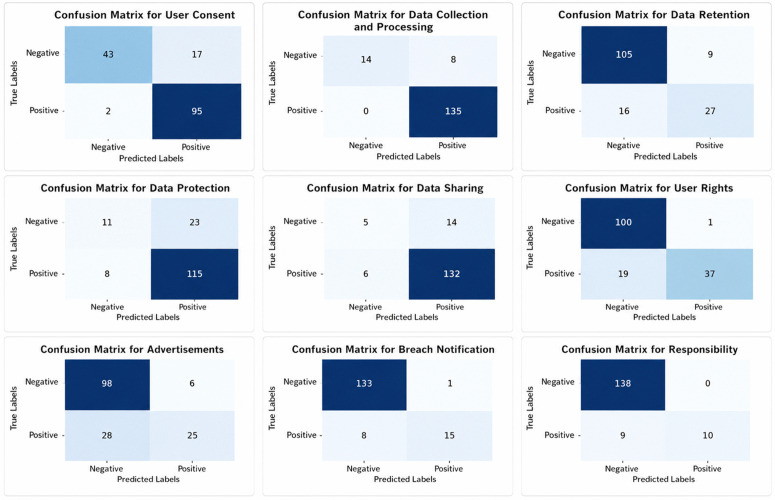
Confusion matrices for RF model in Phase 1 (baseline with imbalanced dataset).

Fig 9 illustrates the confusion matrices of the RF model, which had the best performance. The results show that the model performs well in predicting the majority classes, such as Data Collection and Processing, Data Sharing, and Data Protection. These categories have a higher number of true positive predictions compared to false negatives, indicating the model’s ability to correctly identify instances belonging to these classes. However, the model’s performance is less accurate for minority classes like Responsibility and Breach Notification. In the confusion matrix for Responsibility, the model predicts 138 instances as true negative and only 10 as true positive, with 9 being false negative. Similarly, for Breach Notification, the model predicts 133 instances as true negative and only 15 as true positive, with 8 being a false negative. The closeness between the number of true positive and false negative suggests that the model has difficulties correctly identifying instances of these minority classes. In addition, Advertisement Label was the label most frequently falsely predicted by the model due to the nature of the Advertisement label, which contains the consent to receive advertisements that are closely related to the User Consent label.

### 4.2. Phase 2: Impact of synthetic data augmentation

Following synthetic data generation and class balancing, we re-evaluated the same models to assess the impact of augmentation. Table 6 presents the performance after augmenting the dataset.

**Table 6:** Classifier performance during the second phase with the augmented Saudi Privacy Poli-cy Dataset, assessing the impact of data augmentation on model effectiveness.

Text Representation	Models	Precision	Recall	F-score	Hamming Loss
TF-IDF	LR	0.90	0.89	0.89	0.09
	RF	0.91	0.91	**0.91**	**0.07**
	SVM	0.91	0.92	**0.91**	**0.07**
Word2vec	LR	0.85	0.85	0.85	0.12
	RF	0.90	0.88	0.89	0.09
	SVM	0.85	0.86	0.86	0.12

The results showed that all models demonstrated significant performance improvement after data augmentation and balancing. Among the models, SVM with TF-IDF achieved the best performance (F-score: 0.91), closely followed by RF with TF-IDF (F-score: 0.91). Word2Vec representations yielded relatively improved performance but remained lower than TF-IDF. These results suggest that the TF-IDF representation, which captures the importance of words based on their frequency and uniqueness, remains highly effective for the classification task even after dataset augmentation. Both SVM and RF with TF-IDF achieved the lowest Hamming Loss (0.07), demonstrating substantial improvement in minimizing prediction errors after data augmentation compared to Phase 1 (0.13).

**Fig 10:**
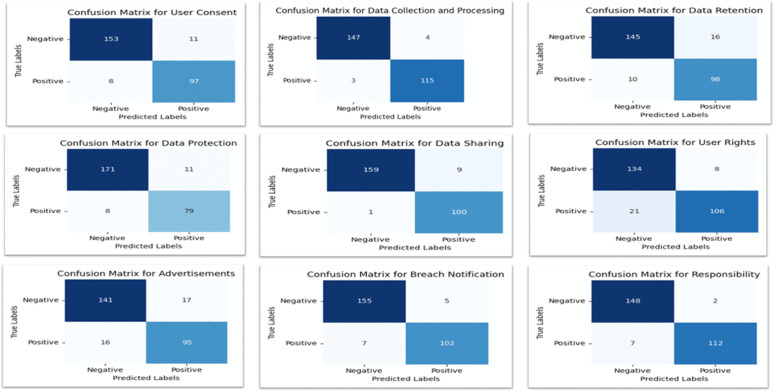
Confusion matrices for SVM model in Phase 2 (after synthetic data augmentation).

Fig 10 illustrates the confusion matrices of the SVM model, which had the best performance. The results show that the model performs well in predicting the majority classes, such as Data Collection and Processing, Data Sharing, and Data Protection, with improved balance across all categories.

However, the model’s performance showed significant improvement for minority classes like Responsibility and Breach Notification. In the confusion matrix for Responsibility, the model predicts 112 instances as true positive and 148 as true negative, with only 7 being false negative and 2 being false positive. Similarly, for Breach Notification, the model predicts 102 instances as true positive and 155 instances as true negative, with reduced false negatives compared to Phase 1. The improved balance between true positive and false negative suggests that the model has overcome previous difficulties in correctly identifying instances of these minority classes. In addition, while the false negatives for Advertisements have improved, the false positives have increased, making it still the worst label in prediction due to the semantic overlap with the User Consent label.

### 4.3. Phase 3: Effect of merging data protection and data sharing labels

In the third phase, we evaluated the decision to merge Data Protection and Data Sharing labels based on the extensive vocabulary overlap observed during data analysis. Table 7 presents the performance after label merging.

**Table 7:** Classifier performance during the third phase after merging Data Protection and Data Sharing labels.

Text Representation	Models	Precision	Recall	F-score	Hamming Loss
TF-IDF	LR	0.92	0.87	0.90	0.08
	RF	0.92	0.91	**0.92**	**0.07**
	SVM	0.92	0.92	**0.92**	**0.07**
Word2vec	LR	0.87	0.85	0.86	0.12
	RF	0.91	0.89	0.90	0.09
	SVM	0.88	0.86	0.87	0.11

The results showed that the performance of the models improved after merging Data Protection and Data Sharing labels. RF and SVM models achieved the highest precision, recall, and F-score values of 0.92 when using TF-IDF, followed by the LR model with an F-score of 0.90. The Hamming Loss remained at 0.07 for both RF and SVM with TF-IDF, maintaining the low error rate achieved in Phase 2 while improving overall classification performance through label merging.

**Fig 11:**
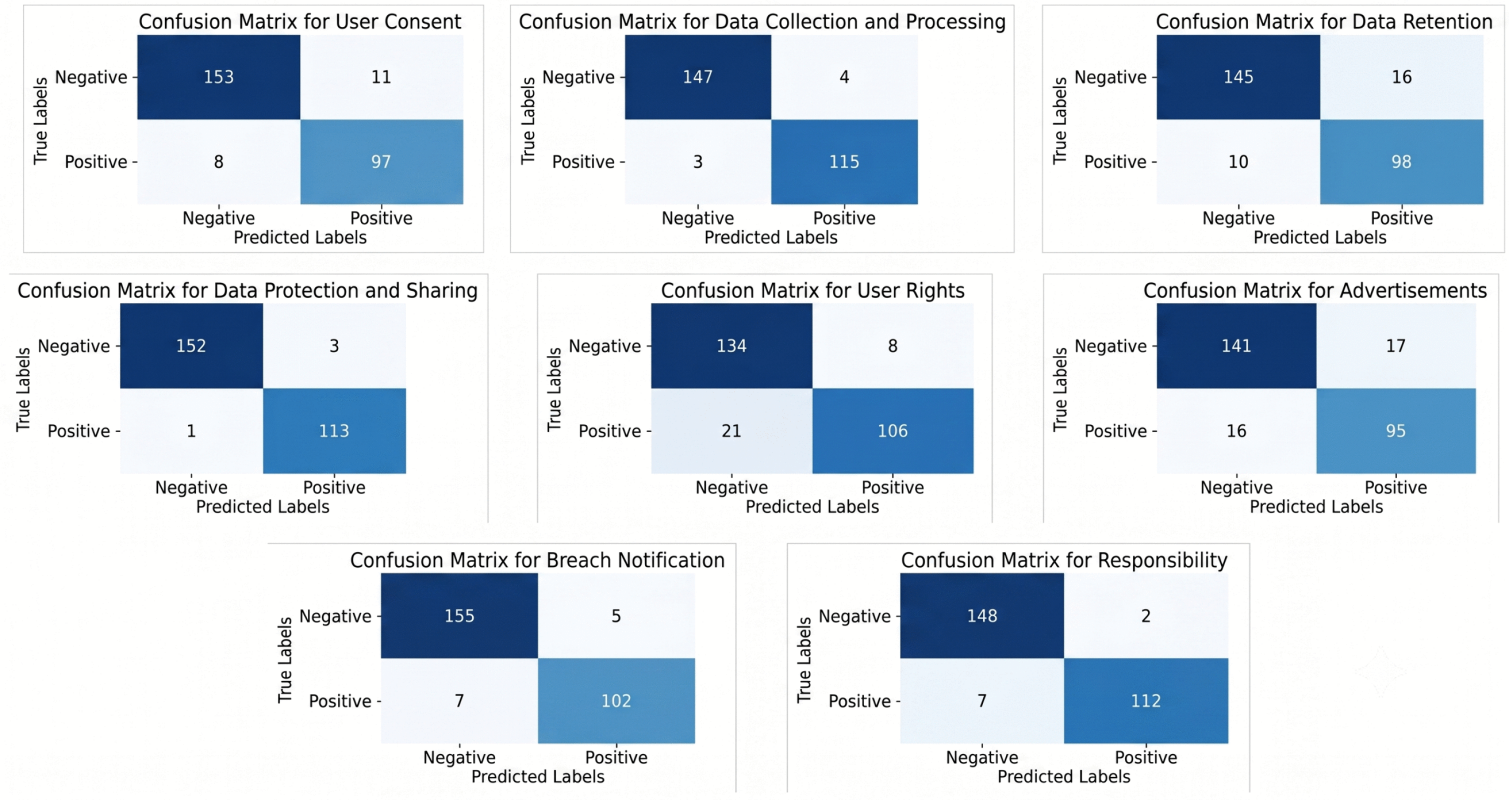
Confusion matrices for SVM model in Phase 3 (after merging Data Protection and Data Sharing labels).

Fig 11 illustrates the confusion matrices of the SVM model, which achieved the best results in the third phase after merging the Data Protection and Data Sharing categories. The merged category shows a well-balanced distribution of true positives and true negatives, with 143 instances correctly predicted as positive and 152 instances correctly predicted as negative. The false positives and false negatives are reduced to only 1 and 3 instances, respectively. These results confirm the decision to merge the Data Protection and Data Sharing labels, as it led to improved model performance.

The remaining categories still retained reasonable performance. However, Advertisement continues to be the most challenging category for the model, with the highest number of false positives at 17, although having 95 true positives and 141 true negatives.

### 4.4. Phase 4: Transformer-based models

For Transformers, introducing an overlap value of 100 between text chunks has improved the performance of both CamelBERT and AraBERT transformers, illustrated in Table 8.

**Table 8:** Classifier performance during the fourth phase (Transformer-Based Models).

Transformer	Overlap Value	Precision	Recall	F-score
CamelBERT	Without	0.91	0.84	0.88
	100	0.87	**0.92**	0.90
AraBERT	Without	0.88	0.90	0.89
	100	**0.91**	0.89	0.90

CamelBERT has shown a notable increase in recall, from 0.84 to 0.92, indicating its enhanced ability to identify relevant information across the segmented text chunks. In addition, the overall F-score has improved from 0.88 to 0.90 although a minor trade-off in precision goes from 0.91 to 0.87.

On the other hand, AraBERT also has shown a significant increase in precision, from 0.88 to 0.91, indicating its enhanced ability to identify relevant information across the segmented text chunks. However, the recall decreased by 1%. This inverse relationship between precision and recall led to an improved F-score by 1%. We iterated the run of transformer scripts 5 times and observed that if the precision improved, the recall decreased, consequently resulting in an approximately constant F-score. These results validate the effectiveness of our methodology in handling long privacy policies by leveraging transformer-based techniques and implementing an overlapping strategy.

**Fig 12:**
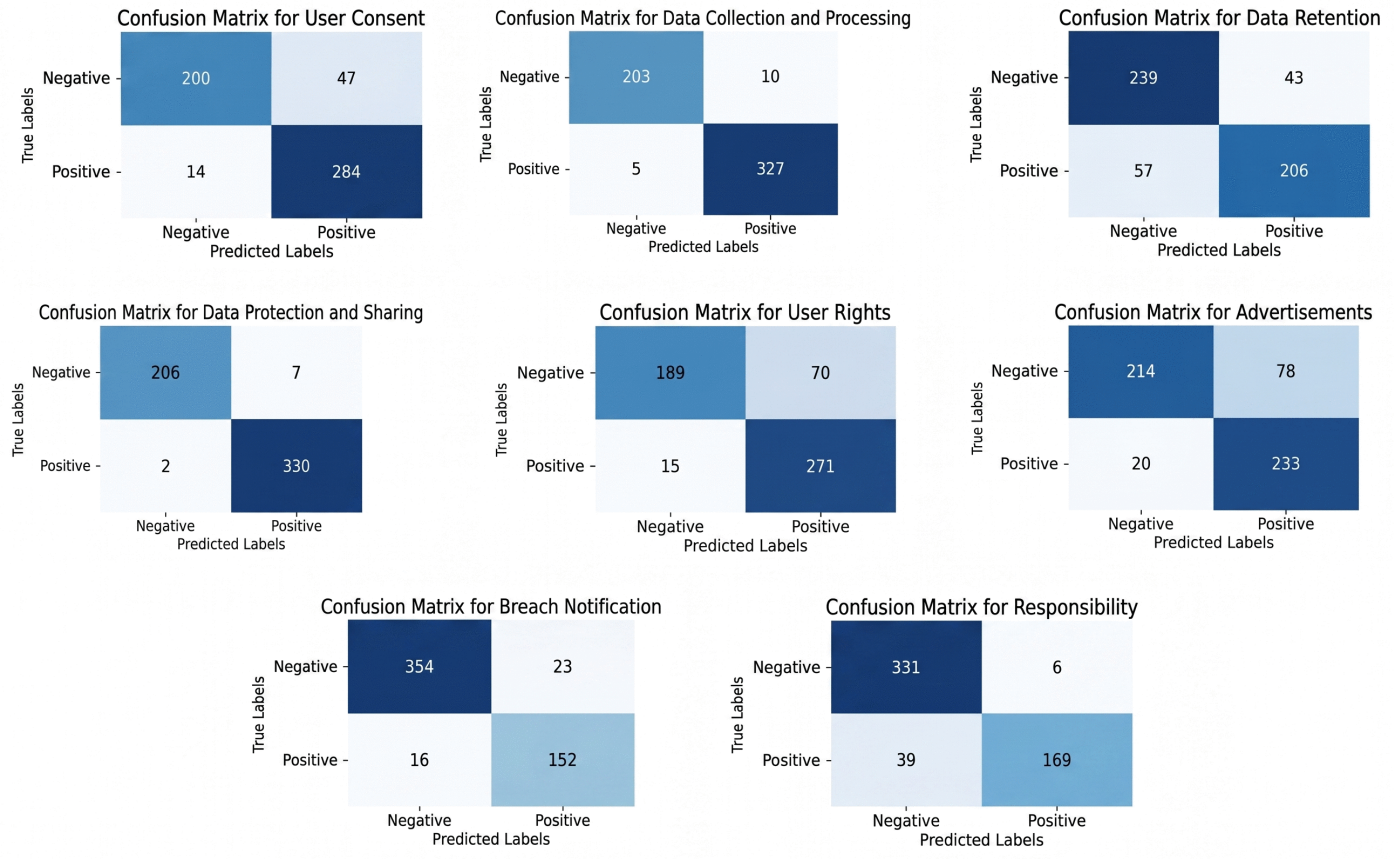
Confusion Matrices for the CamelBERT Transformer with 100 Tokens Overlap.

The confusion matrix of CamelBERT illustrated in **Fig 12**, which achieved the best results in the fourth phase.

## 5. Discussion

Our experimental approach demonstrates how systematic dataset refinement and model selection contribute to effective Arabic privacy policy classification according to PDPL compliance. It is important to note that the proposed approach focuses on detecting PDPL-related categories rather than assessing full legal compliance.

The traditional machine learning model experiments highlighted the importance of feature representation, with TF-IDF consistently outperforming Word2Vec. This aligns with studies such as [23,24] that emphasize the robustness of frequency-based features for MLC tasks. Our findings also support the effectiveness of using the BR technique to address MLC challenges by converting them to multiple binary classifications, as in [26,27].

The observed challenges with minority classes underscored the critical impact of class imbalance on classification performance. Synthetic data augmentation proved effective in addressing this limitation, resulting in improvements across all models, particularly for previously underrepresented categories. This validates the use of LLM-generated data for enhancing dataset balance and model generalizability, with confusion matrices showing more consistent prediction distributions across all PDPL categories.

The decision to merge Data Protection and Data Sharing labels, motivated by extensive vocabulary overlap identified during data analysis, further enhanced performance while reducing task complexity. This highlights the importance of data analysis and understanding dataset characteristics in improving classification effectiveness.

While transformer models demonstrated competitive performance with overlapping text chunks to handle long documents, traditional ML approaches—particularly SVM with TF-IDF—achieved better results.

### Statistical Validation and Model Robustness

To assess the reliability of the reported results, we conducted additional statistical analyses comparing the proposed model against baseline methods, including LR, RF, and SVM, each evaluated using TF-IDF and Word2Vec representations. All models were evaluated using 7-fold cross-validation.

The proposed model achieved a mean micro-F1 score of 92.01% with a standard deviation of 0.39%, indicating low variability across validation folds. The corresponding 95% confidence interval ([91.65%, 92.37%]) suggests a stable performance estimate. In comparison, the best-performing baseline model (Random Forest with TF-IDF) achieved a mean micro-F1 score of 86.10% with a standard deviation of 0.82% (95% confidence interval: [85.34%, 86.86%]).

Paired t-tests were conducted to compare fold-wise performance between the proposed model and each baseline. All comparisons yielded p-values below 0.001, indicating that the observed performance differences are statistically significant.

In addition to higher average performance, the proposed model demonstrated greater stability, as reflected by a low coefficient of variation (0.43%), indicating consistent performance across different training–testing splits.

To provide a more complete statistical picture, we also report the Macro-averaged F1 score, which weights each label equally and is therefore particularly informative in the presence of class imbalance. For the best-performing model (SVM with TF-IDF, Phase 3), the Macro-F1 score was 92%, compared to the Micro-F1 score of 92.01%. The near equality between Macro-F1 and Micro-F1 suggests that the model performs consistently across both frequent and less frequent labels, indicating strong overall robustness across the PDPL categories.

### Limitations

While our study has made significant progress in automating data privacy compliance with the PDPL, we have encountered some limitations that may affect the tool’s performance. The dataset was initially imbalanced, and although expanded with recent privacy policies and synthetic documents generated using ChatGPT, MLC remains a complex task, and some imbalances among label sets and within labels persist. Additionally, the synthetic policies were shorter than real-world policies, which may reduce their ability to capture the linguistic and structural complexity of longer legal documents. This may affect the generalizability of the model to real-world privacy policies.

Furthermore, while the SVM-based model achieved strong performance in this study, it relies on feature-based representations and therefore has limited capacity to capture rich contextual dependencies. This may affect its ability to fully model highly nuanced or implicitly stated policy language.

Another challenge was organizing the dataset, where each category contains different aspects. Therefore, compliance with one category does not necessarily imply full compliance within that category.

In addition, the current evaluation does not include a strict real-only holdout set, meaning that performance estimates are derived from a dataset that mixes real-world and synthetic privacy policies. While the synthetic data was carefully validated by human annotators, its inclusion may inflate performance metrics relative to a purely real-world deployment scenario. Future work should evaluate the system on a held-out set of real privacy policies collected after the training period to provide a more conservative and ecologically valid performance estimate. Furthermore, temporal robustness has not been tested in this study: the dataset was collected at a specific point in time, and privacy policies are subject to change as organizations update their practices in response to regulatory guidance. Assessing how model performance degrades over time, or with policies written under different drafting conventions, is an important direction for future evaluation.

## 6. Conclusion and future work

In this paper, we developed Mumtathil, an automated tool for assessing Arabic privacy policies’ compliance with the PDPL. By leveraging advanced text classification techniques, including ML and transformer-based models, we demonstrated the effectiveness of automating compliance assessment. We were able to experiment with various techniques, including ML models such as SVM, RF, and LR, as well as transformer-based techniques like AraBERT and CamelBERT. Finally, we selected the SVM classifier as the core component of our tool due to its outperforming results, with an F-score of 92%.

Future work will focus on further expanding and balancing the dataset, refining category definitions, and separating overlapping rights into clearer labels. Exploring whether website behaviors align with their stated policies also presents a valuable direction for assessing real compliance. Extending the methodology to privacy regulations across diverse regions and languages. This would involve integrating multilingual transformer models to accommodate various regulatory frameworks, thus increasing the system’s range of use.

Overall, this study advances automated Arabic privacy policy analysis and provides a practical foundation for scalable PDPL compliance assessment in real-world applications.

## Supporting information

S1 Table Sample of the Saudi Privacy Policy Dataset with English translation. (DOCX)
