# Supplementary material for: Mumtathil: Automatic PDPL compliance identification system of Arabic privacy policies documents
Source: PLoS One. 2026 Jun 10;21(6):e0351074. doi: 10.1371/journal.pone.0351074 (PMC13252743; doi:10.1371/journal.pone.0351074)
Supplement: S1 Table — (DOCX) [file pone.0351074.s001.docx]

Table 1: Sample of the Saudi Privacy Policy Dataset with English Translation

| **Arabic Privacy Policy** | **English Translation** |
| --- | --- |
| البيانات الشخصية التي نجمعها تختلف البيانات التي نجمعها بناءً على الغرض من استخدامها ومع ذلك فيما يلي أمثلة على البيانات التي نجمعها البيانات التي يتم جمعها من خلال نماذج الطلبات أو الاستبيانات أو غيرها من الوثائق أو الاتصالات على سبيل المثال لا الحصر الاسم الكامل وتاريخ الميلاد وأرقام الهوية بيانات عن معاملاتك ومنتجاتك معنا مثل نشاط الحساب واستخدام المنتجات البيانات التي يتم جمعها من خلال استخدامك لخدماتنا أو مواقعنا الإلكترونية أو تطبيقات الهواتف المحمولة مثل ملفات تعريف الارتباط البيانات التي يتم تبادلها أثناء تواصلنا معك مثل طلبات خدمة العملاء والمرئيات التي تصلنا منك إما من خلال مواقعنا الإلكترونية أو تطبيقات الهاتف المحمول أو حسابات وسائل التواصل الاجتماعي أو أي قنوات نستخدمها للتواصل مع العملاء الأساس القانوني وأغراض جمع واستخدام بياناتك الشخصية نقوم بجمع واستخدام البيانات وفقاً لما هو متطلب بناء على طبيعة الخدمات والأعمال في معظم الحالات يتضمن أساسنا القانوني وأغراضنا على سبيل المثال لا الحصر الامتثال لأية التزامات ومتطلبات صادرة عن الجهات القانونية والتنظيمية داخل المملكة العربية السعودية السماح لنا بتقديم المنتجات أو الخدمات لحمايتك من الاحتيال عن طريق إجراء عمليات التحقق من الهوية والائتمان أو التحقق من عدم وجود تعارض تحسين منتجاتنا وخدماتنا وتجربتك المقدمة عبر قنواتنا فهم احتياجاتك كعميل وأهليتك للمنتجات والخدمات الترويج لمنتجات وخدمات استثمارية ومالية جديدة قد تهمك كيف يتم جمع بياناتك الشخصية هناك العديد من نقاط الاتصال التي تساعدنا في جمع بياناتك وبعض الأمثلة عند انشاء العلاقة والاستفادة من الخدمة عندما تستخدم خدماتنا وتصبح عميلاً وتستفيد من منتجاتنا تبادل المعلومات عند إكمال نموذج ورفعه على موقعنا الإلكتروني أو تطبيقات الهاتف المحمول أو البريد الإلكتروني أو منشورات وسائل التواصل الاجتماعي أو عبر الاتصال بمركز العملاء أو المشاركة في الحملات الترويجية التفاعل وسائل التواصل الاجتماعي أي منشورات أو تفاعل أو تواصل تجريها معنا بشكل مباشر على قنوات التواصل الاجتماعي نمط التصفح بيانات حول كيفية استكشافك لموقعنا على الانترنت وتوقيت زيارتك وأنواع متصفح الإنترنت وكيف تمت إحالتك إلى موقعنا الاستطلاعات استطلاعات الويب عبر الإنترنت التي تمكننا من جمع الآراء بشأن المواضيع مثل ما يعجبك وما لا يعجبك بشأن شكل ومظهر موقعنا الإلكتروني وتطبيقات الأجهزة المحمولة ملاحظاتك القيمة تمكننا من تحسين جودة التجربة التي نقدمها لك كمنظمة منك شخصياً أو من وكيلك المعتمد أومن ممثلك القانوني في الحالات المقبولة قانونياً من أطراف ثالثة ومصادر عامة وهذا يشمل الجهات الحكومية وشركات ووكالات المعلومات الائتمانية تخزين بياناتك الشخصية ستكون بياناتك الشخصية محتفظ بها طالما كان ذلك ضروريًا لتحقيق الغرض الذي تم جمعها من أجله محتفظ بها داخل المملكة العربية السعودية محتفظ بها لتلبية أي متطلبات قانونية وتنظيمية حماية بياناتك الشخصية نحن نضمن حماية بياناتك بشكل مناسب من خلال استخدام الإجراءات والاحتياطات الأمنية المادية والتقنية والإدارية والإجرائية اللازمة لمنع الوصول غير المصرح به أو الجمع أو الاستخدام أو الكشف أو النسخ أو التعديل أو التخلص منها نحمي بياناتك بطريقة تتوافق مع أنظمة ولوائح حماية البيانات ذات العلاقة السارية بها في المملكة العربية السعودية ومع ذلك فإن الإنترنت ليس آمنًا بشكل كامل الإفصاح عن بياناتك الشخصية قد نشارك بياناتك مع أي من الأطراف المذكورة أدناه أي محكمة أو أي جهة حكومية أو تنظيمية للامتثال لأي التزامات ومتطلبات صادرة عن جهات قانونية وتنظيمية داخل المملكة العربية السعودية أي وكيل تحصيل ديون أو جهة مرخصة بتقديم خدمة المعلومات الائتمانية أو شركة تأمين أي عضو في المجموعة أو أي مزود خدمة مرتبط بأي عضو بغرض تزويدك بالمنتجات أو الخدمات وتحسين منتجاتنا وخدماتنا وتجربتك عبر قنواتنا والترويج لمنتجات وخدمات الاستثمار المالي الجديدة التي قد تهمك قد يتم التواصل معك و أو إرسال إشعارات إليك عبر قنوات مختلفة مثل الرسائل القصيرة و أو البريد الإلكتروني و أو الهاتف فيما يتعلق بمنتجاتنا وخدماتنا التغييرات على إشعار الخصوصية نحتفظ بالحق في تعديل إشعار الخصوصية هذا في أي وقت يشير تاريخ نفاذ إشعار الخصوصية كما هو مذكور أدناه إلى أحدث تاريخ تمت فيه مراجعة إشعار الخصوصية هذا أو تعديله يتيح لك التحقق من تاريخ النفاذ أدناه تحديد ما إذا كانت هناك تغييرات منذ آخر مرة قمت فيها بمراجعة إشعار الخصوصية لذلك ننصحك بالتحقق بشكل دوري على إشعار الخصوصية هذا لضمان اطلاعك على النسخة المحدثة استخدام ملفات تعريف الارتباط من أجل تحسين تجربة عملائنا تستخدم مواقعنا الإلكترونية تقنية قياسية تسمى ملفات تعريف الارتباط لجمع معلومات حول كيفية استخدام مواقعنا الإلكترونية والتي قد تتضمن بياناتك يعد استخدام ملفات تعريف الارتباط أمرًا ضروريًا لتشغيل مواقعنا الإلكترونية اتصل بنا في حالة وجود أي أسئلة يمكنك التواصل معنا من خلال أي من فروعنا والبريد الإلكتروني والمحادثة الفورية ووسائل التواصل الاجتماعي أو من خلال موقعنا الإلكتروني وتطبيق الهاتف المحمول | The personal data we collect varies depending on its intended purpose. However, below are examples of the types of data we collect:  Data collected through application forms, surveys, or other documents and communications, including but not limited to full name, date of birth, and identification numbers.  Information about your transactions and interactions with our products, such as account activity and product usage.  Data collected through your use of our services, websites, or mobile applications, including cookies.  Information exchanged during our communication with you, such as customer service requests and media you share with us through our websites, mobile applications, social media accounts, or other communication channels we use.  We collect and use data as required by the nature of our services and business operations. In most cases, our legal basis and purposes include but are not limited to compliance with any legal and regulatory obligations issued by authorities in Saudi Arabia, enabling us to provide products or services, protecting you from fraud by conducting identity and credit verification or conflict-of-interest checks, improving our products, services, and the experience provided across our channels, understanding your needs as a customer and assessing your eligibility for products and services, and promoting new financial and investment products and services that may be of interest to you.  There are multiple touchpoints through which we collect data. Some examples include when you establish a relationship with us and benefit from our services as a customer, when you fill out and submit a form on our website, mobile applications, email, social media posts, customer service calls, or participation in promotional campaigns, social media interactions, including any posts, engagements, or communications you initiate with us through our social media channels, browsing behavior, including data on how you explore our website, visit timings, browser types, and referral sources, online surveys that allow us to collect feedback on topics such as what you like or dislike about our website and mobile applications. Your valuable feedback helps us improve the quality of our services, and information collected directly from you, your authorized representative, or legally accepted third parties and public sources, including government agencies, credit information companies, and related institutions.  Your personal data will be retained as long as necessary to achieve the purposes for which it was collected. It will be stored within Saudi Arabia and retained to fulfill any legal and regulatory requirements.  We ensure appropriate protection of your data through the use of physical, technical, administrative, and procedural security measures to prevent unauthorized access, collection, use, disclosure, copying, modification, or disposal. We protect your data in compliance with the relevant data protection laws and regulations in Saudi Arabia. However, it is important to note that the internet is not completely secure.  We may share your data with any of the following entities: any court or government or regulatory authority to comply with legal and regulatory obligations in Saudi Arabia, any debt collection agency, licensed credit information service provider, or insurance company, and any member of our corporate group or any service provider associated with a group member for the purpose of providing you with products or services, improving our offerings, and promoting new financial investment products that may interest you.  You may also be contacted or receive notifications through various channels, including SMS, email, or phone calls, regarding our products and services.  We reserve the right to modify this privacy notice at any time. The effective date of this notice, as mentioned below, indicates the most recent revision or modification date. Checking the effective date allows you to determine whether any changes have been made since your last review.  Therefore, we encourage you to periodically review this privacy notice to ensure that you are informed of any updates.  To enhance our customers' experience, our websites use a standard technology called cookies to collect information about how our websites are used. This may include your personal data.  The use of cookies is essential for the operation of our websites.  If you have any questions, you can contact us through any of our branches, email, live chat, social media, website, or mobile application. |
